# *Fructobacillus fructosus* OS-1010 strain stimulates intestinal cells to secrete exosomes that activate muscle cells

**DOI:** 10.1007/s10616-023-00610-1

**Published:** 2024-01-26

**Authors:** Riku Kashiwagi, Miyako Udono, Yoshinori Katakura

**Affiliations:** 1 Graduate School of Systems Life Sciences, Kyushu University, 744 Motooka, Nishi-ku, Fukuoka, 813-0395 Japan; 2 Faculty of Agriculture, Kyushu University, 744 Motooka, Nishi-ku, Fukuoka, 813-0395 Japan

**Keywords:** *Fructobacillus*, Intestinal cells, Muscle cells, Exosome

## Abstract

*Fructobacillus* is a lactic-acid bacterium recently identified in fructose-rich environments. *Fructobacillus* is also known to exhibit unusual growth characteristics due to an incomplete gene encoding alcohol/acetaldehyde hydrogenase, which results in an imbalance in the nicotinamide adenine mononucleotide (NAD^+^)/NADN levels. Recently, the addition of d-fructose to the culture medium of *Fructobacillus* strains increased the intracellular nicotinamide mononucleotide (NMN) content. In the present study, we evaluated the functionality of *Fructobacillus* that produces high levels of NMN, using one substrain (*Fructobacillus fructosus* OS-1010). Therefore, in this study, we examined its functionality in the interaction between intestinal cells and muscle cells. The results showed that supernatant derived from intestinal epithelial cells (Caco-2 cells) treated with *F. fructosus* OS-1010 activated muscle cells (C2C12 cells). Further analysis revealed that Caco-2 cells treated with *F. fructosus* OS-1010 secreted exosomes known as extracellular vesicles, which activated the muscle cells. Furthermore, pathway analysis of the target genes of miRNA in exosomes revealed that pathways involved in muscle cell activation, including insulin signaling and cardiac muscle regulation, neurotrophic factors, longevity, and anti-aging, can be activated by exosomes. In other words, *F. fructosus* OS-1010 could activate various cells such as the skin and muscle cells, by secreting functional exosomes from the intestinal tract.

## Introduction

*Fructobacillus* is a recently discovered lactic-acid bacterium and unlike conventional lactic-acid bacteria, it is known to grow poorly on glucose and has a preference for oxygen (Endo et al. 2018). *Fructobacillus* have been isolated from fructose-rich environments, including flowers, fruits, fermented fruits and the guts of insects that feed on fructose-rich plants. *Fructobacillus* is known to exhibit unusual growth characteristics due to an incomplete gene encoding a bifunctional alcohol/acetaldehyde hydrogenase, which results in an imbalance in the nicotinamide adenine mononucleotide (NAD^+^)/NADH levels. Interestingly, a recent study reported that strains belonging to the genus *Fructobacillus* use d-fructose as an electron acceptor in aerobic lactic acid fermentation, and the addition of d-fructose to the culture medium increases the intracellular nicotinamide mononucleotide (NMN) content. Sugiyama et al. (2021) successfully isolated a strain of *Fructobacillus* with high NMN production. *Fructobacillus fructosus* OS-1010 used in this study is a substrain that produces high levels of NMN, and the purpose of this study was to explore its functionality.

NAD^+^ is one of the cofactors involved in redox reactions in living organisms (Imai 2009; Imai and Guarente 2016). It is synthesized intracellularly from nicotinamide (NAM) and NMN via the salvage pathway. In particular, NAMPT, which synthesizes NAD^+^ from NMN, is the rate-limiting enzyme in this pathway. Recently, a series of reports have focused on its pharmacological effects on various diseases, including cardiovascular disease, Alzheimer’s disease, and diabetes (Yoshino et al. 2011; Poddar et al. 2019). If the amount of intracellular NAD^+^ can be increased, either directly or via conversion of NMN, it is believed that NAD^+^ can activate the longevity gene Sirtuins, which acts as a cofactor and may ultimately lead to anti-aging of the organism (Imai and Guarente 2014). NAD^+^ is known to have such a wide range of functions, and *F. fructosus* OS-1010, which produces NMN and NAD^+^, would have additional functions as a lactic acid bacteria in addition to those of NMN it produces.

Previous studies have found that *F. fructosus* OS-1010 directly acts on myotubular C2C12 cells and activates their mitochondria. In this study, in order to clarify the function of *F. fructosus* OS-1010 as a food, we tried to examine its indirect function on C2C12 cells via the intestinal cells.

## Materials and methods

### Cell culture

The human colorectal cancer cell line Caco-2 (ATCC, Manassas, VA, USA) and murine skeletal myoblasts C2C12 (Riken Bioresource Center, Tsukuba, Japan) were cultured in Dulbecco’s modified Eagle’s medium (DMEM) (Nissui, Tokyo, Japan) containing 10% heat-inactivated fetal bovine serum (FBS, Capricorn Scientific GmbH, Ebsdorfergrund, Germany) at 37 °C in 5% CO_2_. C2C12 cells (2.0 × 10^5^ cells/mL in a 6-well dish) were cultured for 48 h and the medium was replaced with DMEM containing 2% horse serum (HS) (Thermo Fisher Scientific, Waltham, MA, USA) to induce differentiation. After another 24 h, the medium was replaced with DMEM containing 2% HS. The medium was replaced every 2 days and used for experiments 10 days after the induction of differentiation.

### Lactic acid bacteria

Substrain OS-1010 of *Fructobacillus fructosus*, which produces high levels of NMN and NAD^+^, was provided by Osaka Soda Co. Ltd. (Osaka, Japan). The OS-1010 was heated at 65 °C for 30 min and subsequently spray-dried. This dead bacterial powder contained 2.7 µmol of NMN and 14.2 μmol of NAD per gram of dried bacterial weight. The supernatant of Caco-2 cells treated with *F. fructosus* OS-1010 was prepared by centrifugation at 3000×*g* at 10 min.

### Reverse transcriptase-quantitative polymerase chain reaction (RT-qPCR)

RNA was prepared from cells using the High Pure RNA Isolation Kit (Roche Diagnostics GmbH, Mannheim, Germany) according to the manufacturer’s protocol. RT-qPCR was performed using the GoTaq 1-Step RT-PCR System (Promega, Madison, WI, USA) and Thermal Cycler Dice Real Time System TP-800 (Takara, Shiga, Japan). The samples were analyzed in triplicate. The PCR primer sequences used were as follows: mouse *β-actin* forward primer 5′-TGGCACCCAGCACAATGAA-3′ and reverse primer 5′-CTAAGTCATAGTCCGCCTAGAAGCA-3′; mouse *Sirt1* forward primer 5′-GCAGACGTGGTAATGTCCAAACAG-3′ and reverse primer 5′-ACATCTTGGCAGTATTTGTGGTGAA-3′; mouse *Tfam* forward primer 5′-CATTTATCTATCTGAAAGCTTCC-3′ and reverse primer 5′-CTCTTCCCAAGACTTCATTTC-3′; mouse brain-derived growth factor (*Bdnf*) forward primer 5′-GTCAAGTTGGGAGCCTGAAATAGTG-3′ and reverse primer 5′-AGGATGCTGGTCCAAGTGGTG-3′; and mouse *MyoD* forward primer 5′-ATGAGGCCTTCGAGACGCTC-3′ and reverse primer 5′-CAGAGCCTGCAGACCTTCGA-3′. *β-actin* was used as a housekeeping gene. Samples were normalized and analyzed by the ΔΔCT method (Ishibashi et al. 2023).

### Mitochondria

Cells were stained with 250 nM MitoTracker Red CMXRos (Thermo Fischer Scientific) at 37 °C for 30 min and subsequently with 200 nM MitoTracker Green FM (Thermo Fischer Scientific) at 37 °C for 30 min. Finally, cells were stained with Hoechst 33,342 (Dojindo, Kumamoto, Japan) at 37 °C for 30 min. Stained cells were analyzed using an IN Cell Analyzer 2200 (Cytiva, Tokyo, Japan) to quantitatively determine the number, area, and activity of mitochondria, and the images were analyzed using the IN Cell Investigator high-content image analysis software (Cytiva) (Inotsuka et al. 2021).

### Exosome isolation and treatment

Firstly, Caco-2 cells (1.4 × 10^5^ cells/mL) were cultured in DMEM containing 10% exosome-depleted FBS (System Bioscience, Mountain View, CA, USA) and 100 µg/mL OS-1010 for 24 h. After culture, the MagCapture Exosome Isolation Kit PS ver. 2 (FUJIFILM Wako Pure Chemical Corp.) was used to isolate exosomes from the medium of Caco-2 cells, according to the manufacturer’s instructions. The amount of exosomes used for each experiment was prepared as a protein equivalent and measured using a MicroBCA Protein Assay Kit (Thermo Fisher Scientific Inc.) (Sugihara et al. 2019; Ogawa et al. 2021).

### miRNA microarray assay

The expression profiles of miRNAs in the exosomes were evaluated by microarray analysis using a 3D-Gene Human miRNA Oligo chip (Toray, Kanagawa, Japan). MiRNA preparation and subsequent operations were performed by Kamakura Techno-Sciences, Inc. (Kanagawa, Japan). After global normalization of the miRNA expression levels, we calculated the ratios of each miRNA for comparison between the control and experimental samples. We then established criteria for the regulated miRNA: (upregulated miRNA) ratio ≥ 1.5-fold (Bolstad et al. 2003). The miRNA target genes were predicted using TargetScan (https://www.targetscan.org/vert_80/, accessed February 1, 2023). We then used the tools and data provided by Database for Annotation, Visualization, and Integrated Discovery (DAVID, http://david.abcc.ncifcrf.gov, accessed on February 20, 2023) to identify significantly enriched pathways (Huang et al. 2009a, 2009b; Inotsuka et al. 2021; Ishibashi et al. 2023).

### Statistical analysis

All experiments were repeated at least three times, and representative data are shown. The results are shown as the mean ± standard error. Multiple comparisons between groups were performed using a one-way ANOVA with Tukey’s post-hoc test. Statistical significance was defined as p < 0.05 when compared to the control (*p < 0.05; **p < 0.01; ***p < 0.001).

## Results

### *Fructobacillus fructosus* strain OS-1010 activates muscle cells via intestinal cells

**Fig. 1:**
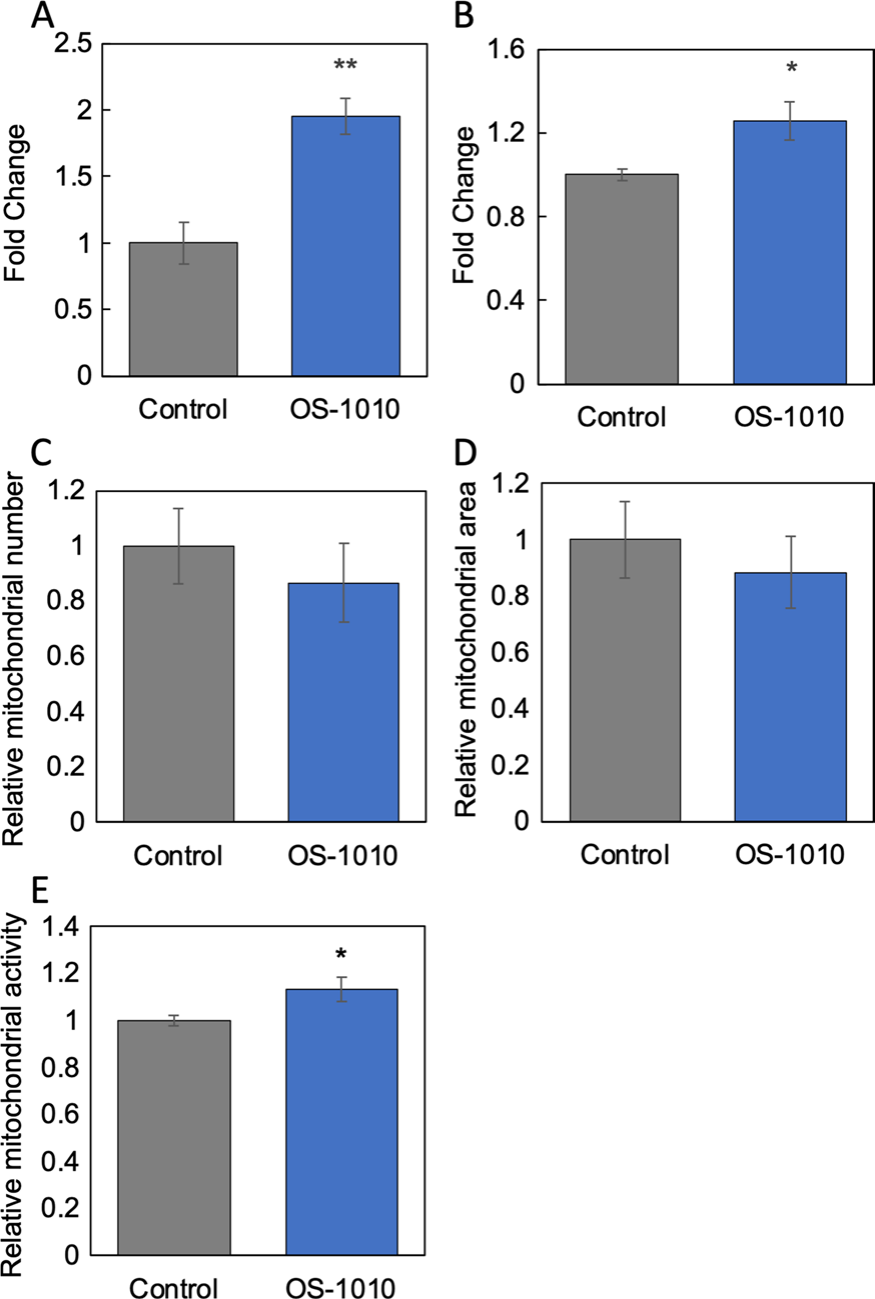
OS-1010 activates muscle cells via intestinal Caco-2 cells. Culture supernatants were prepared from the Caco-2 cells treated with PBS (Control) and 100 µg/mL OS-1010. Then, 10% of the culture medium of C2C12 cells was replaced with this supernatant, and cells were further cultured for 24 h. The expression levels of *Sirt1* (**A**) and *Tfam* (**B**) were tested by RT-PCR. Mitochondrial number (**C**), area (**D**) and activity (**E**) in C2C12 cells added with supernatant were evaluated using the IN Cell Analyzer 2200. The results are shown as the mean ± standard error. Multiple comparisons between groups were performed using one-way ANOVA with Tukey’s post-hoc test. Statistical significance was defined as p < 0.05 when compared to control (*p < 0.05; **p < 0.01; ***p < 0.001)

We firstly examined the possibility that *Fructobacillus fructosus* strain OS-1010 (*F. fructosus* OS-1010) may activate the interaction between intestinal cells and muscle cells. Therefore, we prepared culture supernatants of OS-1010-treated Caco-2 cells and evaluated whether the addition of supernatants to C2C12 cells, a differentiated myotube cell line, affected their phenotypes. The results showed that the culture supernatant of OS-1010-treated Caco-2 cells induced the expression of mitochondria-related genes and activated mitochondria. Sirt1 is known to be involved in mitochondrial biosynthesis, and mitochondrial transcription factor A (Tfam) is a known transcription factor that functions in the mitochondria (Chandrasekaran et al. 2019). As shown in Fig. 1A and B, the supernatant derived from OS-1010-treated Caco-2 cells significantly activated *Sirt1* and *Tfam* expression. These results showed that OS-1010 induced the secretion of secretory factors from Caco-2 cells that activate muscle cells.

The effect of this supernatant on the mitochondria of C2C12 cells was analyzed by measuring the number, area and activity of intracellular mitochondria using mitochondria-specific fluorescent reagents (MitoTracker Red CMXRos and MitoTracker Green FM) and an IN Cell Analyzer 2200. The results showed that the supernatant did not significantly affect the number and area of mitochondria in C2C12 cells but did significantly increase mitochondrial activity (Fig. 1C–E). These results indicate that OS-1010 acts on Caco-2 cells and induces the secretion of factors that activate the mitochondria of muscle cells.

### Supernatant of OS-1010-treated Caco-2 cells induced the expression of marker genes in muscle cells

**Fig. 2:**
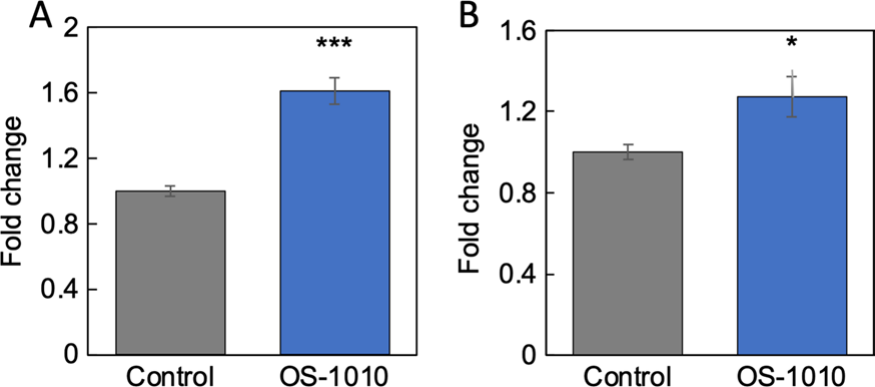
Supernatant of OS-1010-treated Caco-2 cells induced the expression of marker genes in muscle cells. Culture supernatants were prepared from the Caco-2 cells treated with PBS (Control) and 100 µg/mL OS-1010. Then, 10% of the culture medium of C2C12 cells was replaced with this supernatant, and the cells were further cultured for 24 h. The expression levels of *Bdnf* (**A**) and *MyoD* (**B**) were tested by RT-PCR. The results are shown as the mean ± standard error. Multiple comparisons between groups were performed using one-way ANOVA with Tukey’s post-hoc test. Statistical significance was defined as p < 0.05 when compared to control (*p < 0.05; **p < 0.01; ***p < 0.001)

Next, the effects of these supernatants on the expression of marker genes in muscle cells were analyzed. Brain derived neurotrophic factor (Bdnf) is a type of myokine secreted by muscle cells, and MyoD is a known regulator of muscle differentiation (Huang et al. 2022). As shown in Fig. 2A and B, the supernatant derived from OS-1010-treated Caco-2 cells significantly increased the expression of *Bdnf* and *MyoD* in C2C12 cells. This result indicates that the supernatant may promote C2C12 differentiation and activate cell–cell interactions through myokine secretion.

### Exosomes derived from the supernatant of OS-1010-treated Caco-2 cells activate mitochondria in C2C12 cells

**Fig. 3:**
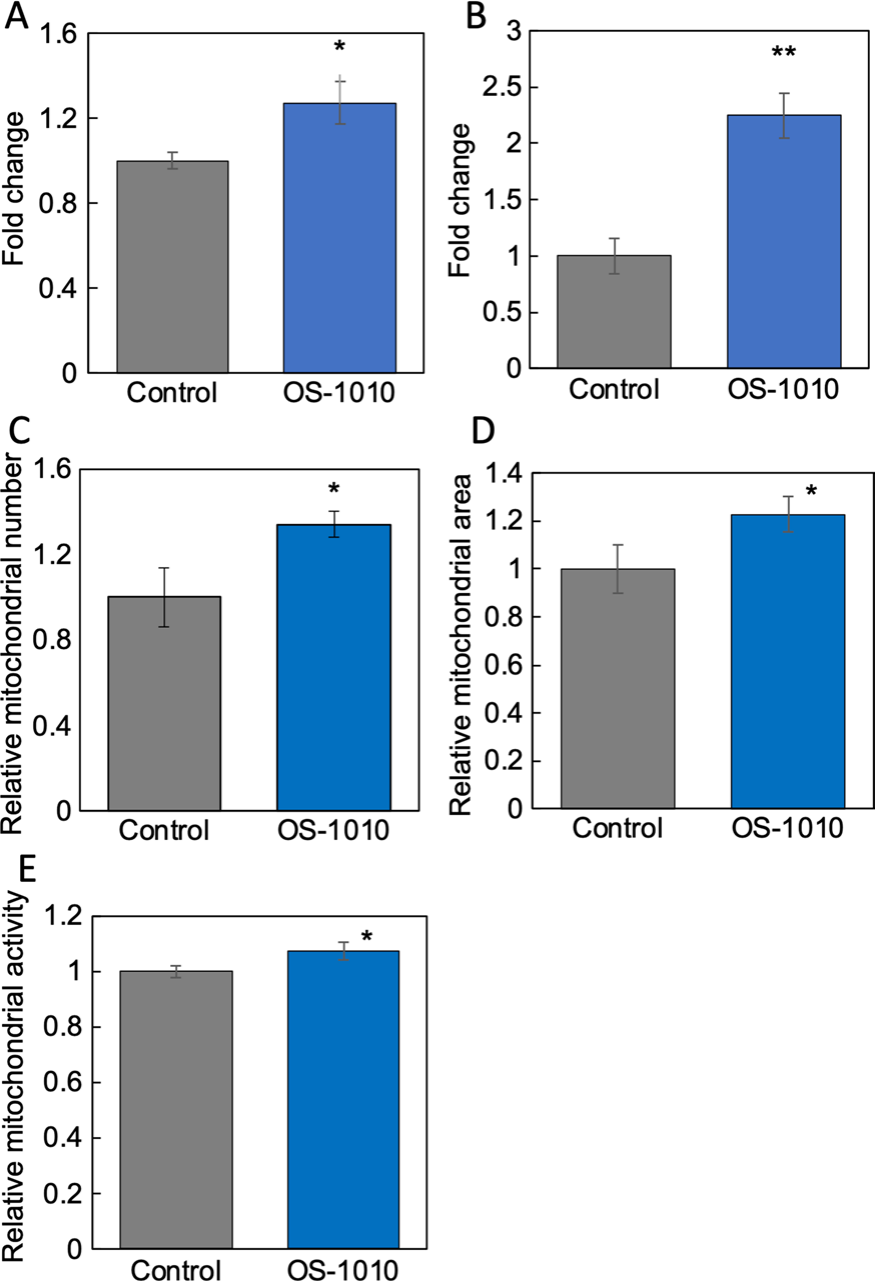
Exosomes derived from OS-1010-treated Caco-2 cells activated C2C12 cells. Exosomes were prepared from the supernatant of Caco-2 cells treated with PBS (Control) and 100 µg/mL OS-1010. These exosomes (180 ng/mL) were added to C2C12 cells, and the cells were further cultured for 24 h. The expression levels of *Sirt1* (**A**) and *Tfam* (**B**) were tested by RT-PCR. Mitochondrial number (**C**), area (**D**) and activity (**E**) in C2C12 cells added with exosomes were evaluated using the IN Cell Analyzer 2200. The results are shown as the mean ± standard error. Multiple comparisons between groups were performed using one-way ANOVA with Tukey’s post-hoc test. Statistical significance was defined as p < 0.05 when compared to control (*p < 0.05; **p < 0.01)

The above results showed that OS-1010-treated Caco-2-derived culture supernatants activated muscle cells. Therefore, we focused on the exosomes in the supernatant and evaluated their functionality. Exosomes were prepared from the supernatant of OS-1010-treated Caco-2 cells using MagCapture Exosome Isolation Kit PS ver. 2 and their functionality against C2C12 cells was evaluated (Sugihara et al. 2019). The results showed that exosomes significantly enhanced *Sirt1* expression, a gene involved in mitochondrial biosynthesis, and *Tfam*, a mitochondrial transcription factor, in C2C12 cells (Fig. 3A and B). Furthermore, exosomes enhanced the number, area, and activity of mitochondria in C2C12 cells (Fig. 3C–E). Unlike the supernatant, the use of exosomes clearly enhanced the effect on C2C12 cells, indicating that exosomes are potent active components secreted by OS-1010-treated Caco-2 cells.

### Exosomes derived from the supernatant of OS-1010-treated Caco-2 cells induced the expression of marker genes in muscle cells

**Fig. 4:**
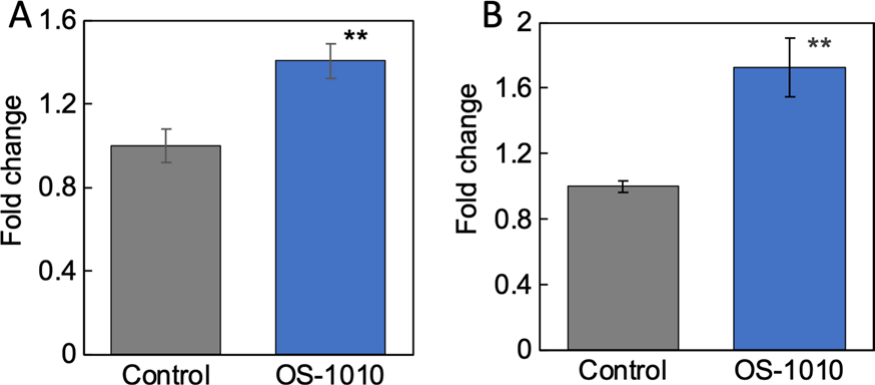
Exosomes derived from OS-1010-treated Caco-2 cells induced the expression of marker genes in muscle cells. Exosomes were prepared from the supernatant of Caco-2 cells treated with PBS (Control) and 100 µg/mL OS-1010. Exosomes (180 ng/mL) were added to C2C12 cells, and the cells were further cultured for 24 h. The expression levels of *Bdnf* (**A**) and *MyoD* (**B**) were tested by RT-PCR. The results are shown as the mean ± standard error. Multiple comparisons between groups were performed using one-way ANOVA with Tukey’s post-hoc test. Statistical significance was defined as p < 0.05 when compared to control (**p < 0.01)

Similarly, in the present study, exosomes derived from the supernatant of OS-1010-treated Caco-2 cells significantly enhanced the expression of *Bdnf* and *MyoD* (Fig. 4). Thus, OS-1010-treated Caco-2-derived exosomes may play an important role in the differentiation and activation of muscle cells, and these exosomes may be mediators of various cell–cell interactions that are not limited to intestinal-to-muscle interactions but originate in the gut. The reason why the results of Figs. 3 and 4 are detectable as a clear change compared to the results of Figs. 1 and 2 may be due to the difference in the amount of exosomes used: Figs. 3 and 4 use 180 ng/mL of protein equivalent exosomes, while Figs. 1 and 2 use only about 1 order of magnitude less.

### Molecular basis for the OS-1010-induced interaction between intestinal cells and muscle cells

The aforementioned results indicated that exosomes derived from the supernatant of OS-1010-treated Caco-2 cells activate muscle cells in terms of mitochondrial activation and augmented expression of various marker genes. We then focused on the miRNAs in exosomes derived from OS-1010-treated Caco-2 cells and identified four miRNAs whose expression was significantly altered by OS-1010 treatment (Table 1). miRNAs that showed more than 1.5-fold higher expression compared to the control treatment are shown in Table 1.

**Table 1:** miRNAs highly expressed in exosomes from OS-1010-treated Caco-2 cells

miRNA	Ratio
1419-5p	1.65
6829-5p	1.58
8089	1.57
1202	1.52

After estimating the target genes of these miRNAs, KEGG pathway analyses was performed using DAVID (Table 2). KEGG pathway analysis revealed that these miRNAs significantly affected pathways involved in the maintenance of muscle cell functionality and activation. In particular, the pathways involved in insulin signaling and cardiac muscle regulation are widely affected. Among the miRNAs analyzed, miR-8089 was found to regulate a wide range of pathways involved in the maintenance of muscle function. Activation of these pathways can result in the activation of mitochondria in muscle cells and the promotion of muscle cell differentiation, as described above. The neurotrophin signaling pathway is also activated by these miRNAs, including miR-6829-6p, miR-8089 and miR-1202. This result corresponds to the finding that OS-1010-treated Caco-2-derived supernatants and exosomes augmented Bdnf expression in C2C12 cells (Figs. 2 and 4). Furthermore, the miRNAs identified in this study were found to regulate the pathways involved in longevity, including FoxO, mTOR, and autophagy. *Sirt1*, which was found to be augmented in C2C12 cells by OS-1010-treated Caco-2-derived supernatants and exosomes, is also a known longevity gene, and it could be assumed that these miRNAs enhanced *Sirt1* expression through the activation of the longevity signaling pathway.

**Table 2:** Signaling pathways regulated by miRNAs highly expressed in exosomes from 081010-treated Caco-2 cells

Signaling pathway	KEGG pathway	miRNA	p-value
Insulin signaling	Insulin resistance	1914-5p	0.036
		8089	0.031
		1202	0.043
	Insulin signaling pathway	1914-5p	0.013
		8089	0.001
		1202	0.001
	Pl3K-Akt signaling pathway	1914-5p	0.012
		6829-5p	0.016
		8089	0.016
	FoxO signaling pathway	1914-5p	0.012
Neurotrophin	Neurotrophin signaling pathway	6829-5p	0.026
		8089	0.026
		1202	0.023
Cardiac muscle	Adrenergic signaling in cardiomyocytes	1202	0.008
	Arrhythmogenic right ventricular cardiomyopathy	8089	0.001
	Hypertrophic cardiomyopathy	8089	0.006
		1202	0.036
	Dilated cardiomyopathy	8089	0.011
	Vascular smooth muscle contraction	1202	0.043
Longevity	Longevity regulating pathway	1914-5p	0.002
		1202	0.038
	Longevity regulating pathway-multiple species	1914_5p	0.036
		1202	0.045
	mTOR signaling pathway	1914-5p	0.000
	Autophagy-animal	1914-5p	0.019

## Discussion

In this study, we showed that the *F. fructosus* OS-1010 strain, which produces high levels of NMN and NAD^+^, induces the secretion of exosomes from intestinal cells that can activate muscle cells. It remains to be clarified whether the NMN and NAD^+^ produced by OS-1010 activate intestinal cells or whether OS-1010 activates specific cell–cell interactions with intestinal cells. However, given that relatively high concentrations of NMN (500 µM) have been reported to be necessary to exert an effect on intestinal cells and to validate this effect (Ru et al. 2022), it is highly likely that the OS-1010 itself contributes to the activation of intestinal cells. These should be clarified in the future, but at least from the results of this study, it is clear that OS-1010 treatment induces the secretion of exosomes from intestinal cells that could activate muscle cells.

Our previous studies have shown that food components, such as carnosine and γ-aminobutyric acid (GABA), induce the secretion of exosomes that activate neuronal cells as a result of the activation of intestinal cells; that is, these food components can activate the gut-brain interaction via exosome secretion (Sugihara et al. 2019; Inotsuka et al. 2020, 2021). In other words, exosomes induced secretion by food components could activate interactions between organs and cells. In light of these studies, we can consider the OS-1010 strain as a new food ingredient that can activate the intestine-muscle interactions via exosomes. No reports on food ingredients that can activate intestine-muscle interactions via exosome secretion have been published, and these results indicate the possibility of a novel function for food ingredients via the intestine.

Probiotics such as lactic acid bacteria and bifidobacteria contribute to the suppression of sarcopenia, a disease muscular system, in studies using rodents. Because the skeletal muscle is physiologically distant from the intestinal tract, it is thought that intestinal-derived signals (microbial metabolites, gut peptides, lipopolysaccharides and secretory factors) resulting from intestinal stimulation contribute to the interaction between the intestinal tract and skeletal muscle and regulate muscle function (Giron et al. 2022). The involvement of intestine-derived exosomes, as revealed here, in the activation of the intestine-muscle interaction by probiotics is also expected to be clarified in the future.

## References

[CR1] Bolstad BM, Irizarry RA, Astrand M, Speed TP (2003). A comparison of normalization methods for high density oligonucleotide array data based on variance and bias. Bioinformatics.

[CR2] Chandrasekaran K, Anjaneyulu M, Choi J, Kumar P, Salimian M, Ho CY, Russell JW (2019). Role of mitochondria in diabetic peripheral neuropathy: influencing the NAD(+)-dependent SIRT1-PGC-1alpha-TFAM pathway. Int Rev Neurobiol.

[CR3] Endo A, Maeno S, Tanizawa Y, Kneifel W, Arita M, Dicks L, Salminen S (2018). Fructophilic lactic acid bacteria, a unique group of fructose-fermenting microbes. Appl Environ Microbiol.

[CR4] Giron M, Thomas M, Dardevet D, Chassard C, Savary-Auzeloux I (2022). Gut microbes and muscle function: can probiotics make our muscles stronger?. J Cachexia Sarcopenia Muscle.

[CR5] Huang DW, Sherman BT, Lempicki RA (2009). Bioinformatics enrichment tools: paths toward the comprehensive functional analysis of large gene lists. Nucleic Acids Res.

[CR6] Huang DW, Sherman BT, Lempicki RA (2009). Systematic and integrative analysis of large gene lists using DAVID bioinformatics resources. Nat Protoc.

[CR7] Huang Q, Wu M, Wu X, Zhang Y, Xia Y (2022). Muscle-to-tumor crosstalk: the effect of exercise-induced myokine on cancer progression. Biochim Biophys Acta Rev Cancer.

[CR8] Imai S (2009). The NAD World: a new systemic regulatory network for metabolism and aging–Sirt1, systemic NAD biosynthesis, and their importance. Cell Biochem Biophys.

[CR9] Imai S, Guarente L (2014). NAD and sirtuins in aging and disease. Trends Cell Biol.

[CR10] Imai S, Guarente L (2016). It takes two to tango: NAD(+) and sirtuins in aging/longevity control. NPJ Aging Mech Dis.

[CR11] Inotsuka R, Uchimura K, Yamatsu A, Kim M, Katakura Y (2020). Gamma-aminobutyric acid (GABA) activates neuronal cells by inducing the secretion of exosomes from intestinal cells. Food Funct.

[CR12] Inotsuka R, Udono M, Yamatsu A, Kim M, Katakura Y (2021). Exosome-mediated activation of neuronal cells triggered by gamma-aminobutyric acid (GABA). Nutrients.

[CR13] Ishibashi A, Udono M, Sato M, Katakura Y (2023). Molecular mechanisms for the carnosine-induced activation of muscle-brain interaction. Nutrients.

[CR14] Ogawa M, Udono M, Teruya K, Uehara N, Katakura Y (2021). Exosomes derived from fisetin-treated keratinocytes mediate hair growth promotion. Nutrients.

[CR15] Poddar SK, Sifat AE, Haque S, Nahid NA, Chowdhury S, Mehedi I (2019). Nicotinamide mononucleotide: exploration of diverse therapeutic applications of a potential molecule. Biomolecules.

[CR16] Ru M, Wang W, Zhai Z, Wang R, Li Y, Liang J, Kothari D, Niu K, Wu X (2022). Nicotinamide mononucleotide supplementation protects the intestinal function in aging mice and D-galactose induced senescent cells. Food Funct.

[CR17] Sugihara Y, Onoue S, Tashiro K, Sato M, Hasegawa T, Katakura Y (2019). Carnosine induces intestinal cells to secrete exosomes that activate neuronal cells. PLoS ONE.

[CR18] Sugiyama K, Iijima K, Yoshino M, Dohra H, Tokimoto Y, Nishikawa K, Idogaki H, Yoshida N (2021). Nicotinamide mononucleotide production by fructophilic lactic acid bacteria. Sci Rep.

[CR19] Yoshino J, Mills KF, Yoon MJ, Imai S (2011). Nicotinamide mononucleotide, a key NAD(+) intermediate, treats the pathophysiology of diet- and age-induced diabetes in mice. Cell Metab.

